# Andersen–Tawil Syndrome Is Associated With Impaired PIP_2_ Regulation of the Potassium Channel Kir2.1

**DOI:** 10.3389/fphar.2020.00672

**Published:** 2020-05-15

**Authors:** Reem Handklo-Jamal, Eshcar Meisel, Daniel Yakubovich, Leonid Vysochek, Roy Beinart, Michael Glikson, Julie R. McMullen, Nathan Dascal, Eyal Nof, Shimrit Oz

**Affiliations:** ^1^Sackler School of Medicine, Tel Aviv University, Tel Aviv, Israel; ^2^Heart Center, Sheba Medical Center, Ramat-Gan, Israel; ^3^Neonatology Department, Schneider Children’s Medical Center, Petah-Tikva, Israel; ^4^Baker Heart and Diabetes Institute, Melbourne, VIC, Australia

**Keywords:** Andersen–Tawil syndrome, cardiac arrhythmia, PIP_2_, inward-rectifier potassium channel, slide helix, G-loop, voltage-sensitive phosphatase, BGP-15

## Abstract

Andersen–Tawil syndrome (ATS) type-1 is associated with loss-of-function mutations in *KCNJ2* gene. *KCNJ2* encodes the tetrameric inward-rectifier potassium channel Kir2.1, important to the resting phase of the cardiac action potential. Kir-channels’ activity requires interaction with the agonist phosphatidylinositol-4,5-bisphosphate (PIP_2_). Two mutations were identified in ATS patients, V77E in the cytosolic N-terminal “slide helix” and M307V in the C-terminal cytoplasmic gate structure “G-loop.” Current recordings in Kir2.1-expressing HEK cells showed that each of the two mutations caused Kir2.1 loss-of-function. Biotinylation and immunostaining showed that protein expression and trafficking of Kir2.1 to the plasma membrane were not affected by the mutations. To test the functional effect of the mutants in a heterozygote set, Kir2.1 dimers were prepared. Each dimer was composed of two Kir2.1 subunits joined with a flexible linker (i.e. WT-WT, WT dimer; WT-V77E and WT-M307V, mutant dimer). A tetrameric assembly of Kir2.1 is expected to include two dimers. The protein expression and the current density of WT dimer were equally reduced to ~25% of the WT monomer. Measurements from HEK cells and *Xenopus* oocytes showed that the expression of either WT-V77E or WT-M307V yielded currents of only about 20% compared to the WT dimer, supporting a dominant-negative effect of the mutants. Kir2.1 sensitivity to PIP_2_ was examined by activating the PIP_2_ specific voltage-sensitive phosphatase (VSP) that induced PIP_2_ depletion during current recordings, in HEK cells and *Xenopus* oocytes. PIP_2_ depletion induced a stronger and faster decay in Kir2.1 mutant dimers current compared to the WT dimer. BGP-15, a drug that has been demonstrated to have an anti-arrhythmic effect in mice, stabilized the Kir2.1 current amplitude following VSP-induced PIP_2_ depletion in cells expressing WT or mutant dimers. This study underlines the implication of mutations in cytoplasmic regions of Kir2.1. A newly developed calibrated VSP activation protocol enabled a quantitative assessment of changes in PIP_2_ regulation caused by the mutations. The results suggest an impaired function and a dominant-negative effect of the Kir2.1 variants that involve an impaired regulation by PIP_2_. This study also demonstrates that BGP-15 may be beneficial in restoring impaired Kir2.1 function and possibly in treating ATS symptoms.

## Introduction

Andersen–Tawil syndrome (ATS, also known as long-QT7) is a channelopathy, typically characterized by a triad of symptoms: cardiac arrhythmias, periodic paralysis, and dysmorphic features (Tawil et al., 1994). However, variable symptom severity and incomplete penetrance are associated with the syndrome, in addition to a phenotypic mimicry of catecholaminergic polymorphic ventricular tachycardia (CPVT) characterized by bidirectional ventricular tachycardia (VT) (Barajas-Martinez et al., 2011).

*KCNJ2* encodes the inwardly-rectifying potassium channel Kir2.1, a major component of the cardiac action potential repolarization phase. Pathogenic variants of *KCNJ2* gene account for 60-70% of clinical ATS cases, termed type-1 ATS (Plaster et al., 2001). Most of the pathogenic variants cause Kir2.1 loss-of-function and exert a dominant-negative effect, attributed to trafficking or gating defects (Tristani-Firouzi et al., 2002; Hosaka et al., 2003; Ma et al., 2011). Type-2 ATS is caused by mutations in genes other than *KCNJ2* (Kokunai et al., 2014).

**Figure 1:**
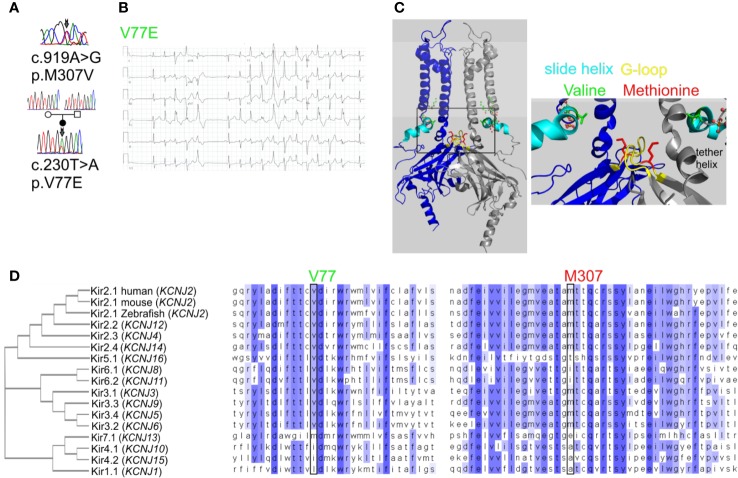
Mutations in Kir2.1 associated with Andersen–Tawil syndrome. **(A)** DNA sequencing of two patients revealed mutations in Kir2.1. **(B)** Representative ECG from the carrier of V77E variant showing bidirectional ventricular tachycardia, prolonged QT interval with ST and T wave abnormalities. **(C)** Left, cartoon presentation of two subunits (blue and gray) out of four, of the inwardly rectifying potassium channel protein (Kir2.2 with PIP_2_, PDB ID: 3SPI (Hansen et al., 2011)). PIP_2_ is in ball-and-stick (atom code: oxygen, red; carbon, green; phosphorous, orange). The N-terminal slide helix is in cyan and the cytoplasmic gate G-loop is in yellow. Side chains as sticks of V75 and M308 in Kir2.2, homologous to V77 and M307 in Kir2.1, are in green and red, respectively. The plasma membrane is in light gray. Right, zoom on the mutated residues’ location. **(D)** Kir channels phylogenetic tree. Amino acid sequence alignments of 15 human Kir proteins, in addition to mouse and Zebrafish Kir2.1.

Kir channels are homotetramers composed of four identical subunits that form a K^+^-selective transmembrane pore. Kir2.1 subunit topology includes two transmembrane domains (TMDs) with the outer and inner helices that form the transmembrane conduction pathway. Regulatory cytoplasmic regions are the N-terminal “slide helix” that lays in parallel to the plasma membrane, and a large cytosolic C-terminal domain (CTD). The assembled CTD structures form a tightening in the cytosolic ion permeation pathway through a gate termed the “G-loop,” aligned below the transmembrane ion permeation pathway (**Figure 1C**).

Kir channels are activated by the phosphoinositide PI(4,5)P_2_ (PIP_2_) (Huang et al., 1998). ATS-associated mutations in Kir2.1 were linked to reduced PIP_2_ affinity (Lopes et al., 2002). The crystal structure of Kir2.2 showed that PIP_2_ interacts with an interface between the inner TMD and the CTD complex, inducing the formation of a cytoplasmic extension of the TMD: the tether helix. The tether helix is pivotal for both the direct binding of PIP_2_ and for the conformational gating transduction (**Figure 1C**) (Hansen et al., 2011; Lacin et al., 2017).

The hydroxylamine derivative BGP-15 is a small molecule that showed beneficial and cytoprotective effects in diabetes, cancer, cardiomyopathy, heart failure, atrial fibrillation (AF), and muscle dysfunction (Gehrig et al., 2012; Literati-Nagy et al., 2014; Sapra et al., 2014; Nascimento et al., 2018). BGP-15 has been well-tolerated in phase I–II human clinical trials as an insulin sensitizer. The putative target pathways include heat-shock proteins induction (Crul et al., 2013), poly ADP-ribose polymerase 1 (PARP-1) inhibition (Racz et al., 2002), and phosphorylation of IGF1R (Sapra et al., 2014). The exact mode of action is not clear, but several reports suggest that BGP-15 modulates plasma membrane composition, dynamics, and fluidity, involving lipid-rafts reorganization (Gombos et al., 2011; Sapra et al., 2014; Balogi et al., 2019).

In the current work, we report two ATS-associated Kir2.1 mutations. Using molecular and electrophysiological methods we show that these mutations are loss-of-function with a dominant-negative effect. We developed a calibrated protocol to asses Kir2.1 functional sensitivity to PIP_2_ using voltage-sensitive phosphatase (VSP) and investigated the effect of BGP-15 on Kir2.1 regulation.

## Materials and Methods

### Clinical and Genetic Assessment

The participants in this study provided written informed consent for both the clinical and genetic studies, which were approved by the Institutional Ethics Committee of the Sheba Medical Center, Tel-Hashomer (approval 0379-13-SMC). Genetic findings were obtained by a sequence screening of the following genes: *KCNQ1*, *HERG*, *SCN5A*, *KCNE1*, *KCNE2*, *KCNJ2*, and *RYR2*. Patients were clinically evaluated by 12-lead electrocardiogram (ECG), exercise testing, and 24-h Holter monitoring.

### Cellular Expression Models

*HEK cells* were maintained in Dulbecco’s modified Eagle’s medium (DMEM) supplemented with 10% fetal bovine serum (FBS), 100 U/ml penicillin, 10 mg/ml streptomycin, and 2 mM L-glutamine (Biological Industries, Kibbutz Beit-Haemek, Israel) at 37°C with 5% CO_2_. Transfections for electrophysiological experiments were using Trans-ITx2 (Mirus, Madison, WI, USA) in 35-mm dishes and biotinylation experiments were using the calcium-phosphate method in 10-cm dishes. 90%–100% confluent dishes were split 1:2 on day 1 and transfected on day 2. On day 3, cells were split on coverslips for patch-clamp experiments and recorded on days 4-5. Biotinylation procedures started on day 4.

*HL-1 cells* were cultured on gelatin/fibronectin pre-coated dishes, in Claycomb media supplemented with 10% FBS, 100 μM norepinephrine (Sigma-Aldrich, St. Louis, Mo, USA), 100 U/ml penicillin, 10 mg/ml streptomycin, and 2 mM L-glutamine (Biological Industries). For imaging, cells were plated on gelatin/fibronectin-coated culture slides (Lab-Tek Nalge Nunc International, IL, USA) and transfected using Lipofectamine 2000 (Thermo Fisher Scientific, Waltham, MA, USA).

*Xenopus laevis oocytes* experiments were approved by Tel-Aviv University Institutional Animal Care and Use Committee (permits M-08-081 and M-13-002). Maintenance and operation of female frogs were essentially as previously described (Oz et al., 2017). Briefly, frogs were maintained at 20 ± 2°C on a 10-h light/14-h dark cycle. Portions of ovary were removed from anesthetized frogs [0.17% procainemethanesulphonate (MS222)]. Oocytes were defolliculated by collagenase, injected with RNA and incubated for 2–3 days at 22°C in NDE solution (in mM: 96 NaCl, 2 KCl, 1 MgCl_2_, 1 CaCl_2_, 5 HEPES, 2.5 pyruvic acid; 50 mg l^−1^ gentamycin).

### DNA Constructs

DNA of GFP was C-terminally fused to the human *KCNJ2* coding sequence (accession number NM_000891.2), following the elimination of *KCNJ2* stop codon and cloned into pcDNA3 vector using restriction enzymes (BamHI-*KCNJ2*-EcoRI-GFP-ApaI). This is the Kir2.1-GFP monomer. DNA sequence encoding the HA-tag (a.a. YPYDVPDYA) was inserted after alanine 115 in the extracellular domain, using a Q5 site-directed mutagenesis kit (New England Biolabs, Ipswich, MA, USA). Point mutations were introduced using PCR [KAPA HiFi HotStart (Kapa Biosciences, Wilmington, MA, USA)]. DNA constructs of Kir2.1 concatenated dimers were prepared using the HA-tagged *KCNJ2* constructs. Kir2.1 WT-WT construct is the WT dimer and WT-V77E, WT-M307V, or WT-R67W construct are mutant dimers. The first Kir2.1 subunit was created with BamHI at the 5′ end, then the stop codon was eliminated and four codons for glycine were inserted, followed by the SalI site at the 3′ end. The second Kir2.1 subunit was WT or mutated, with SalI site at the 5′ end that followed by four codons for glycine, ending with EcoRI site at the 3′. Thus, the linker between two Kir2.1 subunits was composed of (a.a.) GGGGVDGGGG. Kir2.1 dimers between SalI and EcoRI sites were cloned into pcDNA3 vector for mammalian cell’s expression and pGSB vector [modified pGEMHE (Shistik et al., 1998)] for RNA *in-vitro* synthesis. For HEK cells expression danio rerio voltage-sensing phosphatase (dr-VSP) was in pcDNA vector, and for *Xenopus* oocytes expression dr-VSP was in pGEM vector (Hossain et al., 2008).

RNA was transcribed *in vitro* using a standard procedure, from a template cDNA in oocyte expression vectors containing 5′ and 3′ untranslated sequences of *Xenopus* β-globin (Dascal and Lotan, 1992).

### Immunocytochemistry and Imaging

Cells expressing extracellular HA-tag on Kir2.1-GFP were fixed in 1% paraformaldehyde in phosphate buffer saline (PBS) for 10 min to retain GFP fluorescence, without permeabilization, then blocked with CAS blocking agent (Thermo Fisher Scientific) for 30 min at room temperature. Cells were incubated with mouse anti-HA antibody (Millipore, Burlington, MA, USA), washed with PBS, and incubated with Alexa-594 conjugated anti-mouse secondary antibody (ThermoFisher Scientific). Cell nuclei were counterstained with DAPI. Images were captured with an LSM 700 confocal microscope using Zen software (ZEISS, Oberkochen, Germany). Three channels (blue, green, red) were acquired sequentially.

### Biotinylation Assay

Biotinylation of surface proteins was carried out essentially as described (Nof et al., 2019). Briefly, transfected HEK cells were incubated with 0.5 mg/ml EZ-Link Sulfo-NHS-SS-Biotin (Pierce, Rockford, IL, USA) in PBS pH 8, supplemented with 1 mM Ca^2+^ and 0.5 mM Mg^2+^, for 30 min at 4°C. Incubation in 50 mM glycine for 10 min ended the reaction. Cells were scraped, washed and lysed for 30 min at 4°C in (mM): 20 Tris–HCl pH 8, 150 NaCl, 1 EGTA, and 1% NP40 supplemented with 0.5 phenylmethylsulfonyl fluoride (PMSF), protease inhibitor cocktail (Roche, Basel, Switzerland), 25 β-glycerol phosphate, and 1 sodium orthovanadate (Sigma-Aldrich). Equal amounts of total protein were incubated with streptavidin-agarose beads (Pierce) for 2 h at 4°C in a rotating device. Proteins were eluted in sample buffer for 1 h at room temperature, followed by 5-min incubation at 65°C, then resolved by SDS-PAGE and Western blotted.

### Electrophysiology

**Figure 2:**
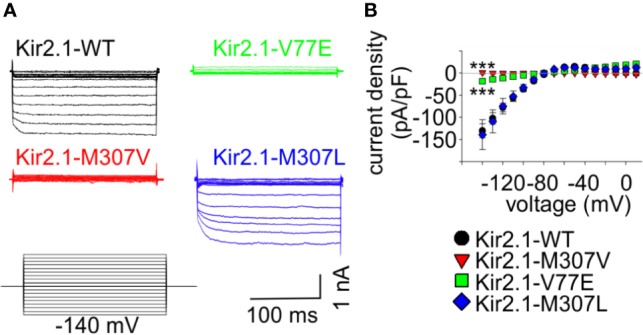
Mutations V77E and M307V induce Kir2.1 loss-of-function. Net Ba^2+^-sensitive currents recorded from HEK cells expressing Kir2.1-GFP (WT, V77E, M307V, M307L) elicited by a voltage-steps protocol. **(A)** Representative currents. **(B)** Average current density (WT, n = 12; V77E, n = 10; M307V, n = 6; M307L, n = 8). Statistical analysis in the voltage step to −140 mV using one-way ANOVA (Dunnett’s test, comparison to WT). M307L, not significant; V77E and M307V, ***p < 0.001.

Whole-cell currents were recorded from HEK cells using the patch-clamp technique, at 23°C. Signals were amplified using an Axopatch 200B patch-clamp amplifier (Molecular Devices, San Jose, CA, USA), sampled at 1 kHz, and filtered with a low-pass Bessel filter at 10 kHz. Data were acquired with DigiData1440A (Molecular Devices). Patch pipettes (Harvard apparatus, Holliston, MA, USA) were pulled with a resistance of 3–5 MΩ. Series resistance was compensated by 80%–90%. The intracellular pipette solution contained (in mM): 130 KCl, 2 MgCl_2_, 4 MgATP, 10 EGTA, 10 HEPES, adjusted with KOH to pH 7.2 (295 mOsm). The external solution contained (in mM): 135 NaCl, 4 KCl, 10 glucose, 10 HEPES, 1.8 CaCl_2_, 1 MgCl_2_, adjusted with NaOH to pH 7.4 (305 mOsm). For Ba^2+^-sensitive currents (**Figure 2**), cells were additionally perfused with the Ba^2+^-containing external solution (in mM): 10 BaCl_2_, 120 NaCl, 4 KCl, 10 glucose, 10 HEPES, 1.8 CaCl_2_, 1 MgCl_2_. The net Ba^2+^-sensitive current was a subtraction of the current evoked in the presence of perfused BaCl_2_ from the current evoked in the standard external solution without BaCl_2_, using the same protocol in one cell.

HEK cells were held at −60 mV in the voltage step protocol (**Figure 2**) and at 0 mV in the voltage ramp protocol. Ramps were set from −120 to 0 mV in 1 s. In the double-ramp protocol (example in **Figure 5A**), a 10-s sweep was followed by 80 s at holding potential before the next sweep. Often, the maximal amplitude of the first ramp in sweeps II–IV was smaller than the maximal amplitude of the first ramp in the previous sweep (probably due to a partial replenishment of PIP_2_ between the sweeps). Using voltage ramps enabled to distinguish Kir2.1 from leak currents that may have developed due to deterioration of the cell during the protocol. When the ramp waveform did not have a typical Kir2.1 rectification the sweep was excluded. VSP effect was quantified from currents elicited by voltage ramps as the ratio between the maximal amplitude at −120 mV after (I2) and before (I1) the depolarizing step, and as % VSP effect according to ((1 − (I2/I1)) * 100). When the amplitude ratio was 1, the VSP effect was 0%, and if VSP activation abolished the current completely, I2/I1 was 0 and the VSP effect was 100%. Normalized ramp-elicited currents were divided by the maximal inward current at −120 mV and plotted against the voltage. The current density was calculated as the current amplitude divided by the capacitance of each cell. Normalized current density was compared to the mean current density of the control group at −120 mV. Cells transfected on the same day and recorded during a 2-day session were considered as one experiment. 50 µM BGP-15 (Cayman Chemical, Ann Arbor, MI, USA) were incubated overnight in cell culture medium and were added to the bath solution during recording.

*Xenopus* oocytes were injected with RNAs of Kir2.1 concatenated dimers (0.05, 0.1, or 1 ng of the WT dimer, as indicated, and 1 ng of the mutant dimers) and VSP (10 ng). Currents were recorded using the two-electrode voltage clamp (TEVC) technique. Oocytes were held at −30 mV and current–voltage relationships were produced by voltage ramps from −120 to 30 mV in 2 s. Bath solution contained (in mM): 24 KCl, 72 NaCl, 1 CaCl_2_, 1 MgCl_2_, and 5 HEPES, in pH = 7.4. Naïve cells’ endogenous, depolarization-induced, inward currents (possibly *via* sodium or stretch-activated ion channels) were partially blocked by 50 µM Gd^3+^ (Baud et al., 1982; Yang and Sachs, 1989). The net Kir2.1 currents were obtained by subtraction of the mean current obtained with the same VSP activation protocol from four naïve oocytes from the same batch of oocytes in the same experiment or in the presence of 50 µM Gd^3+^. VSP effect was quantified from currents elicited by voltage ramps as the ratio between the maximal amplitude at −80 mV. The reversal potential, V_rev_, was the potential at which the net current was zero. The rectification index was calculated by the division of the current 50 mV positive to V_rev_ by the current 50 mV negative to V_rev_.

### Software and Data Analysis and Presentation

The structure of Kir2.2-V75E was generated by PyMOL (Molecular Graphics System, Version 2.0 Schrödinger) based on the crystal structure of Kir2.2 channel (PDB ID:3SPI). The rotamer with minimal steric clashes was selected. Subsequently, the energy minimization procedure was conducted by YASARA force field server default values (http://www.yasara.org) (Krieger et al., 2009).

DNA chromatogram was visualized by BioEdit. Multiple DNA sequence alignments were using CLUSTAL Omega 1.2.4. Western blot bands were quantified using ImageJ (NIH, United States). Figures were prepared using CorelDraw 2018 (Corel Corp., Ottawa, Canada). SigmaPlot 13 and 14 (Systat Software, CA, USA) and Prism (GraphPad, San Diego, CA, USA) were used for graph preparation and statistical assays. Currents were analyzed using Clampfit 10.7.

Data were presented as mean ± standard error or box plots indicating 25–75 percentiles (box borders), median (black line), mean (dotted red line), and 5–95 percentiles (whiskers). Statistical tests were performed on raw data. Two-group comparisons were performed using unpaired t-test if the data passed the Shapiro-Wilk normality test, otherwise, Mann–Whitney rank-sum test was used. Multiple group comparisons were done using one-way ANOVA, followed by Dunnet’s tests for normally distributed data and Kruskal-Wallis one-way ANOVA on ranks followed by Dunn’s test, otherwise. Two-way ANOVA with repeated measures was performed to compare current courses in voltage ramp protocol, between groups. ***, p < 0.001; **, p < 0.01; *, p < 0.05; ns, not significant.

## Results

### Kir2.1 Variants V77E and M307V Are Associated With ATS

We have previously described a case of familial almost incessant polymorphous and bidirectional VT. Association of the disease with mutations in several calcium-handling related genes was excluded (Nof et al., 2004). Years later, a larger gene screening detected a c.919A > G substitution in the *KCNJ2* gene encoding the inward-rectifier potassium channel Kir2.1. This missense mutation led to a substitution of methionine at position 307 to valine (p.M307V) in the Kir2.1 protein (**Figure 1A**). Shortly after the report, one family member had a syncope while driving, and implantable cardioverter defibrillator (ICD) was implanted. Symptomatic arrhythmia events were not documented since then. Another family member was implanted with an ICD due to her ventricular arrhythmias. A few years later, she had a ventricular fibrillation (VF) storm which the ICD was able to terminate. All other family members described in the previous report (Nof et al., 2004) remained asymptomatic and were not implanted with ICD despite the heavy arrhythmic burden.

In another case, a 35-year-old woman with multiple premature ventricular contractions (PVCs) and non-sustained ventricular tachycardia (NSVT) was genetically and clinically evaluated. She reported a lower limb muscle weakness. A thigh-muscle biopsy, performed at another institute, was normal. ECG recording showed polymorphic and bidirectional VT at rest and exercise. Missense variation c.230T > A in *KCNJ2* was detected, leading to substitution of valine 77 to glutamic acid (p.V77E) in Kir2.1. Both parents did not carry the mutation in *KCNJ2*, and kinship genetic test based on single-nucleotide polymorphism (SNP) confirmed parenthood. Thus, V77E mutation is a *de-novo* mutation in the proband (**Figures 1A, B**). She developed tachycardia-induced cardiomyopathy with 40% left ventricular ejection fraction (LVEF). An ablation attempt was aborted due to the polymorphic nature of her PVCs, and an ICD was implanted. She did not receive any ICD shocks during follow up.

Following the association of the *KCNJ2* mutations with cardiac arrhythmia symptoms, ATS type-1 was diagnosed in the carriers of both mutations. The clinical manifestations included ventricular arrhythmias but without periodic paralysis or facial/skeletal dysmorphic features that have been previously reported, with a variable penetrance, in other ATS cases.

Residue M307 is located at the G-loop in Kir2.1 C-terminus. Residue V77 is located at the regulatory slide helix in Kir2.1 N-terminus (**Figure 1C**). Both residues are conserved among species and different inward-rectifier potassium channels. The hydrophobic valine 77 is highly conserved among 11 out of 15 *KCNJ* genes, and in all 15 *KCNJ* genes this position is occupied by hydrophobic residues. The hydrophobic methionine 307 is conserved in 8 out of 15 *KCNJ* genes (**Figure 1D**).

### Both V77E and M307V Cause Kir2.1 Loss-of-Function Without Affecting Cell Surface Expression

In order to study the functional properties of the mutants, Kir2.1-GFP constructs were expressed in HEK cells, and currents were measured using whole-cell patch-clamp technique. During a voltage-step protocol, cells expressing Kir2.1-GFP WT showed large inward voltage-dependent currents at voltages negative to −80 mV that were blocked by 10 mM Ba^2+^. The net Ba^2+^-sensitive currents were inwardly rectifying with a reversal potential close to the calculated K^+^ equilibrium potential (according to the calculated Nernst potential of −90 mV and liquid junction potential of 3.9 mV, the calculated reversal potential of K^+^ is ~−86 mV), and small outward currents were recorded with voltage steps between −80 to −20 mV (**Figure 2**).

Expression of the Kir2.1-GFP V77E and M307V mutants produced almost no Ba^2+^-sensitive currents, indicating that these are loss-of-function mutations. The substitution of M307 with the hydrophobic residue leucine (M307L) did not affect the current density and rectification that were similar to that of Kir2.1-GFP WT (**Figure 2**).

**Figure 3:**
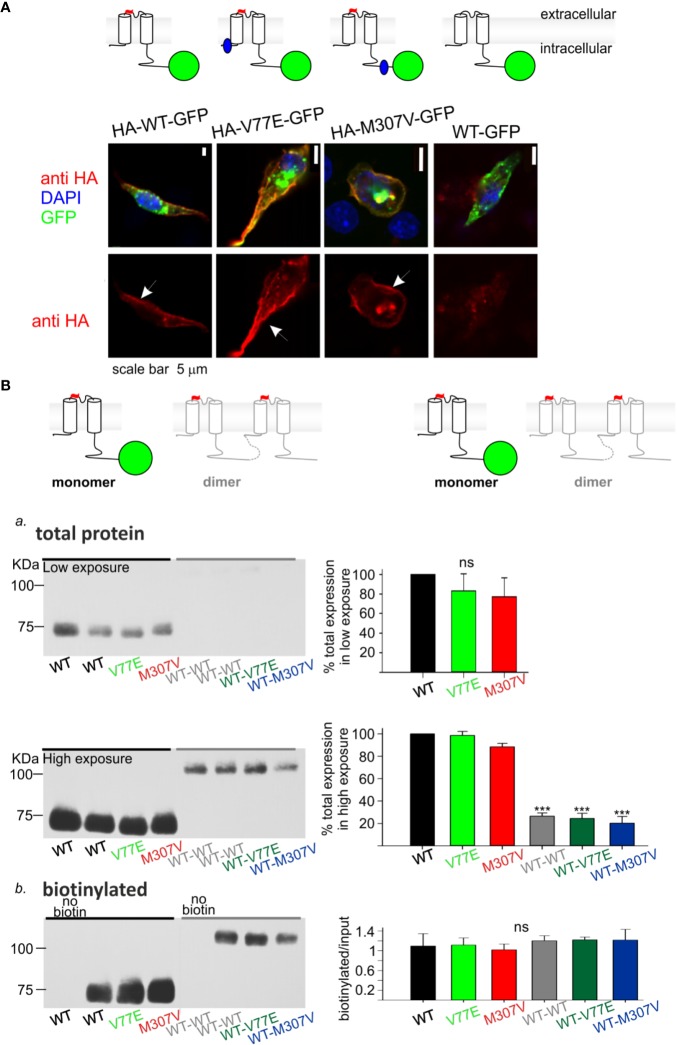
Loss-of-function Kir2.1 mutations express on the cell surface. **(A)** Immunostaining of HL-1 cells transfected with Kir2.1-GFP with and without an extracellular HA-tag. Top, an illustration of the Kir2.1 subunit constructs. Cylinders: Kir2.1 transmembrane domains; blue: mutation location; green: GFP; Red: HA-tag. Bottom, HA-tag was immunostained (red), expressed GFP was detected (green) and the nucleus was stained with DAPI (blue). The three optical channels are superimposed (upper panel) and the red channel is also shown separately (lower panel). Arrows point to the cell surface signal. **(B)** Biotinylation of HEK cells transfected with Kir2.1-GFP monomers and Kir2.1 dimers (WT and mutant) as illustrated above and detected with the HA-tag antibody. Left, representative blots from one experiment. Right, normalized signals. *a*. Total Kir2.1 protein level. Total expression was normalized to WT in the same experiment. Low film exposure was used to quantify the difference in Kir2.1 monomers’ level within a dynamic range (upper panel, N = 4). Statistical analysis: one-way ANOVA. The differences between Kir2.1 monomers and dimers were shown in high film exposure of the blot, where Kir2.1 monomer signal was saturated (lower panel, N = 3). Statistical analysis: one-way ANOVA (Dunnett’s test, comparison to WT Group). *b*. biotin-labeled Kir2.1 level. As a control for non-specific signals, Kir2.1-GFP WT and WT dimer expressing groups were not incubated with biotin. The amount of protein on the plasma membrane relative to total was quantified by a division of the biotinylated with input level in high exposure (N = 3). Statistical analysis: one-way ANOVA.

To investigate whether protein synthesis and trafficking in these loss-of-function Kir2.1 variants were impaired, two methods were employed: imaging of immunofluorescent Kir2.1 and biochemical detection of cell-surface Kir2.1 by biotinylation (**Figure 3**).

Cultured HL-1 atrial cells (Claycomb et al., 1998) were transfected with the WT and mutated Kir2.1-GFP that included an extracellular HA-tag. We used Kir2.1-GFP WT construct that did not include the extracellular HA-tag, as control. HL-1 cells were gently fixed and immunostained with HA-tag antibody. Green fluorescence following the expression of the Kir2.1-GFP was observed, mainly as aggregates in cytoplasmic organelles (probably the endoplasmic reticulum and Golgi apparatus), but also at the cell periphery. Following immunostaining, red fluorescence was detected at the plasma membrane of cells expressing Kir2.1-GFP WT, V77E, and M307V, but not when the HA-tag was not expressed. When the red and green signals were superimposed, a yellow signal was detected on the cell border, demonstrating the expression of Kir2.1-GFP WT, V77E, and M307V at the cell surface (**Figure 3A**).

Next, HEK cells were transfected with Kir2.1-GFP and incubated with biotin. Biotin-labeled cell-membrane proteins and total protein fractions were analyzed using Western blot. Kir2.1-GFP WT, V77E, and M307V were detected at the cell surface with no significant difference between groups, indicating that V77E and M307V did not affect Kir2.1 cellular expression, or cell surface expression that possibly reflects trafficking [**Figures 3Ba** (upper panel), B*b*].

In order to investigate the functional and biochemical properties of each of the two variants, V77E and M307V, expressed in a heterozygotic context, we used a subunit concatenation strategy where two Kir2.1 subunits were fused as dimers. In the Kir2.1 dimers, the first Kir2.1 subunit was WT and the second subunit was either a WT subunit (“WT dimer”) or a mutated subunit (“mutant dimers”). Kir2.1 subunits were connected with a flexible 10 a.a. linker that included eight glycines. When the same amount of DNA was used for the transfection of Kir2.1-GFP monomers and Kir2.1 dimers, total expression levels of Kir2.1 dimers were 20%–30% of the monomers, with no significant difference between the WT and mutant dimers (**Figure 3Ba**, lower panel, **Supplemental image 1**). The expressed Kir2.1 dimers were exported to the cell surface so that the biotinylated fractions of WT and mutant dimers were not significantly different from each other (**Figure 3Bb**, **Supplemental image 2**). These experiments show that the expression of Kir2.1 dimer on the plasma membrane is reduced compared to Kir2.1-GFP monomers, but the mutations do not affect the surface expression of either Kir2.1 monomers or dimers.

**Figure 4:**
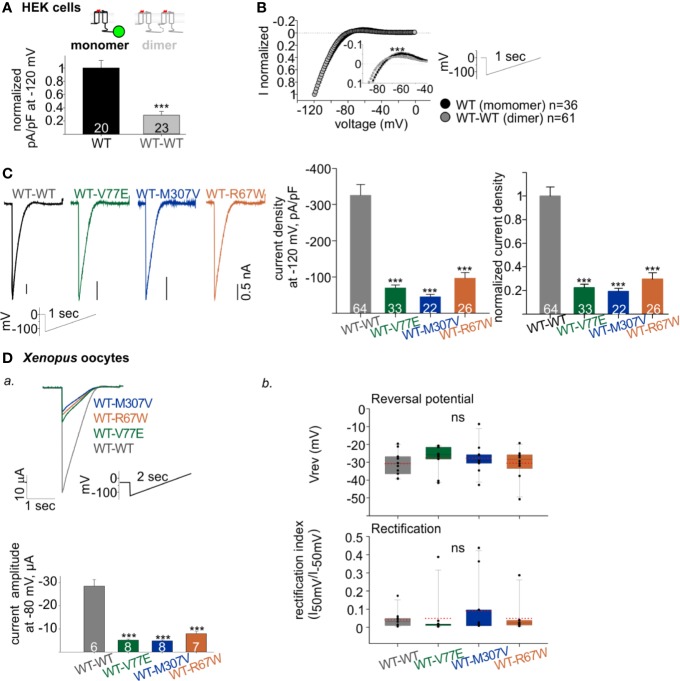
Functional properties of Kir2.1 dimers. **(A)** Normalized current densities of Kir2.1-GFP WT monomer and WT dimers, recorded in HEK cells transfected with equal amounts of DNA. Normalization was performed to the mean current density of Kir2.1-GFP from the same experiment (N = 3). Statistical analysis: t-test. **(B)** Normalized currents (left) elicited by a ramp protocol (right). Every 10^th^ recorded point is presented. This is a summary of all cells from different experiments (N = 7 experiments for Kir2.1-GFP WT monomer and N = 12 for WT dimer). Inset, zoom on the reversal potential and the outward currents. Statistical analysis: two-way ANOVA with repeated measures, p < 0.001. **(C)** Left, representative currents elicited by ramp protocol of HEK cells expressing Kir2.1 dimers. Middle, current densities at −120 mV. Right, current density at −120 mV normalized to mean current density from cells expressing Kir2.1 WT dimers from the same experiment (WT-WT was compared to WT-V77E in N = 7, to WT-M307V in N = 4 and to WT-R67W in N = 6 experiments). Statistical analysis: one-way ANOVA (Dunn’s test, comparison to WT Group). **(D)** Kir2.1 dimers RNA (1 ng/oocyte) was injected in *Xenopus* oocytes and recorded in 24 mM K^+^ bath solution. *a*. Top, representative evoked currents by a voltage ramp protocol. Bottom, average current amplitudes at −80 mV. Statistical analysis: t-test compared to the WT-WT group. N = 1 experiment. *b*. Reversal potentials (Vrev) and rectification index calculated from the current elicited by voltage ramps, n = 10–11, N = 2 experiments. Statistical analysis: one-way ANOVA.

A reduction in WT dimer function correlated with the reduction in its expression level: normalized current density at −120 mV of the WT dimer was 28% ± 5% of the Kir2.1-GFP WT monomer (**Figure 4A**). The normalized current waveform elicited by voltage ramp from −120 to 0 mV showed only a small change in current rectification and selectivity properties of the WT dimer compared to the Kir2.1-GFP WT monomer (**Figure 4B**). These results demonstrate that Kir2.1 subunit concatenation partially affected protein expression, but the cell-surface expressed Kir2.1 composed of the WT dimers had functional properties similar to the Kir2.1-GFP WT monomers.

### Both V77E and M307V Induce a Dominant-Negative Effect on Current Amplitude but Do Not Change the Selectivity and Rectification Properties

The construction of Kir2.1 dimers enabled functional investigation of loss-of-function mutant with a fixed stoichiometry of WT:mutant subunits within a Kir2.1 tetramer. Kir2.1 is expected to be an assembly of 2 dimers, therefore the expression of mutant dimers is expected to form Kir2.1 tetramer composed of two WT and two mutated subunits. As a control, we used the loss-of-function Kir2.1-R67W variant, previously reported as a dominant-negative mutant (Andelfinger et al., 2002) with no effect on cell surface expression (Kalscheur et al., 2014). When each of the mutant dimers were expressed (WT-V77E, WT-M307V, and WT-R67W), typical rectifying Kir2.1 currents were recorded in HEK cells (**Figure 4C**, left). However, when equal amounts of DNA were used for transfection of WT and mutant dimers, the normalized current densities of mutant dimers were 22% ± 3%, 19% ± 2%, and 30% ± 5%, for WT-V77E, WT-M307V, and WT-R67W, respectively, compared to WT dimer (**Figure 4C**, right). The prominent reduction in current indicates that V77E and M307V mutated subunits possibly exert a dominant-negative effect on the functional WT subunits since the reduction in current was beyond their expected relative expression (of 50%) in a Kir2.1 tetramer.

In order to confirm the dominant-negative effect of the mutants, RNA’s of Kir2.1 dimers were injected in *Xenopus* oocytes, and currents were measured. RNA injection in *Xenopus* oocytes enabled to control protein expression and to limit variability between cells, compared to DNA transfection in HEK cells. Similar to HEK cells, oocytes’ current amplitudes in mutants dimers were reduced to 18% ± 1%, 17% ± 1%, and 28% ± 3% of WT dimer, for WT-V77E, WT-M307V, and WT-R67W, respectively (**Figure 4Da**). These results in *Xenopus* oocytes corroborate the putative dominant-negative effect of the loss-of-function Kir2.1 variants M307V and V77E, as shown in HEK cells.

Although a dominant-negative effect is highly plausible, the subunits within the dimer may have additional features not seen in the native channel. Nonetheless, the previously reported dominant-negative variant R67W (Andelfinger et al., 2002) showed a similar reduction in amplitude within the dimer as the two novel mutations V77E and M307V.

Channel selectivity and rectification properties of the mutant dimers were analyzed in *Xenopus* oocytes. The calculation of these parameters in oocytes was reliable due to the large current amplitude compared to HEK cells. Oocytes were perfused with 24 mM K^+^ solution (the cytosolic K^+^ concentration in *Xenopus* oocytes is estimated ~100 mM (Dascal, 1987) and the calculated Nernst potential is ~−36 mV). The reversal potential and rectification index did not show a significant change when mutant dimers were compared to the WT dimer (**Figure 4Db**). Thus, although the dominant-negative mutants strongly reduced the whole-cell conductance in cells expressing Kir2.1 composed of the mutant dimers (WT-V77E, WT-M307V, and WT-R67W), the selectivity and rectification properties of the residual Kir2.1 currents were not affected, compared to Kir2.1 composed of the WT dimers.

### Kir2.1 Mutations V77E, M307V, and R67W Increase Current Sensitivity to PIP_2_ Depletion

We next characterized the functional sensitivity of Kir2.1 to PIP_2_. We depleted PIP_2_ membrane levels by voltage activation of VSP phosphatase that dephosphorylates plasma membrane phosphoinositide at 5′ position upon depolarization (Murata et al., 2005). PIP_2_ depletion *via* VSPs results in a decay of Kir2.1 current (Murata and Okamura, 2007; Sakata and Okamura, 2014; Rjasanow et al., 2015).

**Figure 5:**
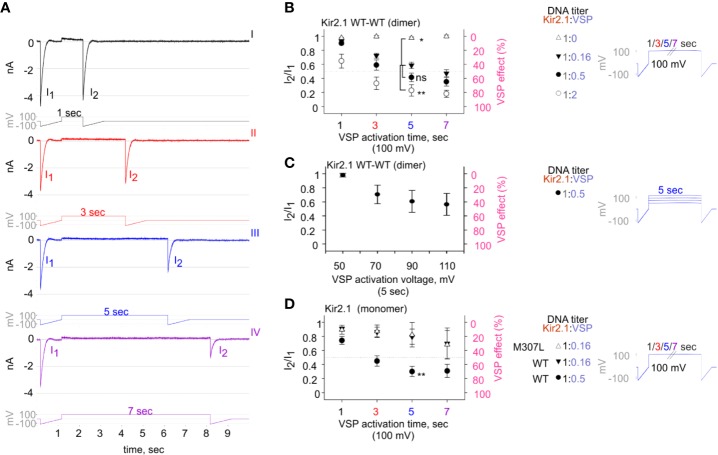
The calibrated VSP activation protocol testing Kir2.1 sensitivity to PIP_2_ depletion by VSP in HEK cells. **(A)** A representative four sweeps (I–IV)-protocol used to quantify Kir2.1 current amplitude reduction following VSP activation by depolarization. Each sweep started with a voltage ramp, followed by a 100-mV voltage step (for 1, 3, 5, or 7 s) and ended with a second voltage ramp. An example of currents elicited in a HEK cell transfected with Kir2.1 WT dimer and VSP in 1:0.16 DNA ratio. **(B–D)** Summary of the maximal amplitude ratio in each sweep after/before VSP activation (left Y-axis), also displayed as % VSP effect (right Y-axis, in pink). Voltage-protocol on the right. **(B)** At DNA ratio Kir2.1:VSP 1:0 (△), n = 10, 6, 4, 3; 1:0.16 (▼), n = 63, 63, 52, 44; 1:0.5 (•), n = 26, 22, 19, 18; 1:2 (○), n = 13, 10, 9, 8 for VSP activation at 100 mV for 1, 3, 5, and 7 s, respectively. Statistical analysis: one-way ANOVA at 5-s VSP activation [comparison to 1:0.16 (▼) group]. **(C)** VSP activation by steps to increasing voltages in each sweep (right). Currents amplitude ratio and the corresponding VSP effect from HEK cells transfected with WT dimer and VSP in 1:0.5 DNA ratio (left). n = 8, 8, 7, 6 for 5-s VSP activation at 50, 70, 90, and 110 mV, respectively. **(D)** Currents amplitude ratio and the corresponding VSP effect in Kir2.1-GFP monomers. At DNA ratio 1:0.16 of Kir2.1-GFP M307L:VSP (△), n = 5, 5, 4, 3; Kir2.1-GFP WT : VSP 1:0.16 (▼), n = 8, 9, 4, 4; Kir2.1-GFP WT : VSP 1:0.5 (•), n = 22, 19, 14, 9 for 1, 3, 5, and 7 s, respectively. Statistical analysis: t-test at 5-s VSP activation of the WT expressing groups, p = 0.004.

We aimed to design a calibrated VSP activation protocol in order to quantify the VSP effect on Kir2.1 currents within a dynamic range. We coexpressed Kir2.1 and VSP in HEK cells and recorded the changes in Kir2.1 current amplitudes following a gradual increase in VSP activation time (**Figures 5A, B**) or voltage (**Figure 5C**). We employed a double-pulse voltage ramp protocol and measured the current amplitudes at −120 mV before VSP activation (I1) and after VSP activation (I2). We then calculated the I2/I1 ratio (left Y-axis, **Figures 5B–D**) and the corresponding % VSP effect (right Y-axis, **Figures 5B–D**). VSP activation by 5-s depolarization to 100 mV was chosen as the standard duration for the following analysis.

VSP activation protocol was additionally calibrated using variable Kir2.1:VSP DNA ratios for transfection. In the group that did not express VSP (Kir2.1:VSP DNA ratio 1:0), the depolarizing pulses did not change the amplitude ratio of I2/I1 and the VSP effect was nearly 0 (**Figure 5B**, empty triangle). Raising the DNA proportion of VSP-to-WT dimers exacerbated the reduction in current amplitudes following VSP activation. After 5 s of depolarization to 100 mV, the second ramp evoked a slight reduction of 2.6% ± 1.7% in the current, in the absence of expressed VSP. When VSP was expressed, current reductions of 41% ± 4.5%, 58% ± 6%, and 77% ± 8% were evoked when the WT dimer:VSP DNA ratios were 1:0.16, 1:0.5, and 1:2, respectively (**Figure 5B**). Augmenting the depolarizing voltage of the 5-s step gradually increased the VSP effect on WT dimer current. VSP effect increased from 2% ± 2.6% to 29.5% ± 13.3%, 39.3% ± 15.6%, and 43.64% ± 15.6% for 50, 70, 90, and 110 mV, respectively (**Figure 5C**).

Current reduction following the VSP activation protocol was also observed in Kir2.1-GFP composed of monomers. The VSP effect was dependent on the transfected DNA proportion Kir2.1:VSP. After 5 s at 100 mV, the VSP effect increased from 20% ± 10% to 70% ± 7% when Kir2.1-GFP WT : VSP DNA ratios were 1:0.16 and 1:0.5, respectively (**Figure 5D**). VSP effect on the functional variant Kir2.1-GFP M307L was similar to that of Kir2.1-GFP WT. After 5 s of VSP activation at 100 mV, the VSP effect on Kir2.1-GFP M307L monomer was 17% ± 17% (Kir2.1:VSP DNA ratio 1:0.16, **Figure 5D**).

**Figure 6:**
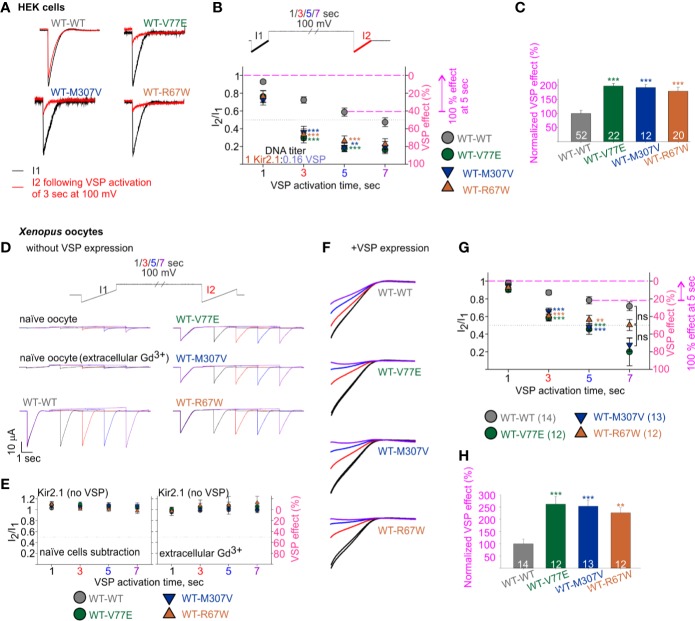
Increased sensitivity of mutant dimers to PIP_2_ depletion using the calibrated VSP activation protocol. **(A–C)** Currents in HEK cells expressing Kir2.1 dimers and VSP. **(A)** Representative ramp currents before (black) and after (red) VSP activation by a 3-s step to 100 mV. Traces of the WT dimer are the same as sweep II in **Figure 5A**. **(B)** Currents amplitude ratio and the corresponding VSP effect recorded from cells transfected with Kir2.1 dimer:VSP DNA ratio of 1:0.16. WT-WT is as summarized in **Figure 5B** (n = 63, 63, 52, 44). WT-V77E, n = 20, 20, 22, 17; WT-M307V, n = 16, 15, 12, 7; WT-R67W, n = 26, 24, 20, 15 for VSP activation by 100 mV step for 1, 3, 5, and 7 s, respectively. Statistical analysis: one-way ANOVA (Dunn’s test, comparison to WT-WT group). At 5-s VSP activation: WT-V77E and WT-R67W, p < 0.001; WT-M307V, p = 0.003. The dashed magenta lines in panel B illustrate the normalizing value for the VSP effect on panel C. **(C)** Normalized VSP effect at a 5-s VSP activation compared to WT dimer. Statistical analysis: one-way ANOVA (Dunn’s test, comparison to WT-WT Group). **(D–H)** Currents in *Xenopus* oocytes expressing Kir2.1 dimers without **(D, E)** and with VSP **(F–H)**. Representative currents in naïve and Kir2.1 dimers expressing oocytes **(D)**, and the average current amplitude ratio and the corresponding VSP effect (left, n = 7–8; right, n = 3–4) **(E)**. **(F)** Representative currents elicited in oocytes using the VSP activation protocol, and following subtraction of the averaged current from naïve cells. I1 and I2 of sweep I are in black, I2 of the sweeps II in red, III in blue, and IV in purple. Current amplitude ratio and the corresponding VSP effect **(G)** and the normalized VSP effect per experiment, at 5-s VSP activation **(H)**. N = 2 experiments. Statistical analysis at 3- and 5-s VSP activation was one-way ANOVA (Dunnett’s test, comparison to WT-WT group). In panel G, at 5-s VSP activation, WT-R67W, p = 0.005. At 7-s VSP activation: statistical analysis was using Kruskal–Wallis one-way ANOVA. In panel H, statistical analysis was using Kruskal–Wallis one-way ANOVA. WT-R67W, p = 0.003. The dashed magenta lines in panel G illustrate the normalizing value for the VSP effect in panel **(H)**.

We then employed the calibrated VSP activation protocol on Kir2.1 mutant dimers, with the parameters that allowed quantification of Kir2.1 current response within a dynamic range. We transfected HEK cells with Kir2.1 WT and mutant dimers, at a Kir2.1:VSP DNA ratio of 1:0.16, and measured the VSP effect on Kir2.1 currents. Using the calibrated VSP activation protocol, with the 5-s step to 100 mV, the VSP effect increased from 41% ± 4.5% in Kir2.1 WT dimers, to 82% ± 3.7%, 81% ± 4.5%, and 74% ± 5.8% in mutant dimers (**Figures 6A, B**), representing a 197% ± 9%, 192% ± 11%, and 179% ± 14% increase in the normalized VSP effect of WT-V77E, WT-M307V, and WT-R67W currents, respectively (**Figure 6C**).

The current amplitude of the Kir2.1 mutant dimers WT-V77E and WT-M307V was 19%–22% of the WT-WT dimer (**Figure 4C**). This reduced current is expected to be the result of a reduced conductance and not due to low protein expression (**Figure 3B**). Thus, the Kir2.1:VSP expression ratio is expected to be similar in mutant compared to WT dimers, suggesting that the strong VSP effect reflects an increased sensitivity to PIP_2_ depletion in Kir2.1 mutant dimers.

We sought to test the calibrated VSP activation protocol in *Xenopus* oocytes (**Figures 6D–H**). We started by applying the VSP activation protocol in naïve *Xenopus* oocytes. We detected time- and voltage-dependent endogenous inward currents. The current amplitude evoked by the voltage ramp increased following depolarization steps, and at −120 mV it often reached a few µA following a 7-s depolarization to 100 mV (**Figure 6D**, a representative naïve oocyte out of six, and one oocyte out of nine in the presence of Gd^3+^). Thus, when we wanted to test VSP effect on Kir2.1 expressing oocytes, we either subtracted the mean current of naïve cells obtained by the VSP activation protocol or applied 50 µM Gd^3+^ in the external solution. Gd^3+^ partially inhibited the increase in the endogenous currents following the depolarization (**Figure 6D**), while Kir2.1 current amplitude and the VSP effect did not change (not shown). When VSP was not expressed in oocytes, the depolarizing pulses did not reduce the currents of WT and mutant dimers, when either method to avoid artifacts by endogenous currents was applied (**Figure 6E**).

We wanted to test whether the reduction in basal current amplitude itself in the mutant dimers’ current (**Figure 4Da**) underlies the difference in VSP effect. Thus, we adjusted RNA amounts of WT and mutant dimes by injecting 10- to 20-fold less RNA of the WT dimer than of mutant dimers, to attain similar amplitudes. When Kir2.1:VSP RNA ratio was 0.1:10 or 0.05:10 in WT dimer and 1:10 in mutant dimers, the average current amplitudes at −80 mV of WT dimer were −10 ± 1.1 µA for 0.1 ng RNA (n = 8) and −1.8 ± 0.11 µA for 0.05 ng RNA (n = 6), while the amplitudes of the mutant dimers were −3 ± 0.3 µA (n = 12), −2.3 ± 0.27 µA (n = 13), and −5 ± 1 µA (n = 12) for WT-V77E, WT-M307V, and WT-R67W, respectively.

Oocytes expressing both VSP and Kir2.1 dimers showed current amplitude decays during the VSP activation protocol (**Figure 6F**). The VSP effect was stronger in all mutant dimers compared to WT dimer, despite the lower Kir2.1:VSP RNA ratio used for WT dimer. The VSP effect, following a 5-s depolarization step to 100 mV, was 21% ± 4% in WT dimer and 54% ± 6%, 52% ± 64%, and 43% ± 65% in mutant dimers (**Figure 6G**), corresponding a normalized VSP effect of 262% ± 631%, 254% ± 625%, and 226% ± 621% in WT-V77E, WT-M307V, and WT-R67W, respectively (**Figure 6H**). These results demonstrate that the mutant dimers WT-V77E, WT-M307V, and WT-R67W have an increased sensitivity to PIP_2_ depletion (and to a similar extent), compared to WT dimers of Kir2.1.

### BGP-15 Decreases Kir2.1 Sensitivity to VSP-Induced PIP_2_ Depletion

**Figure 7:**
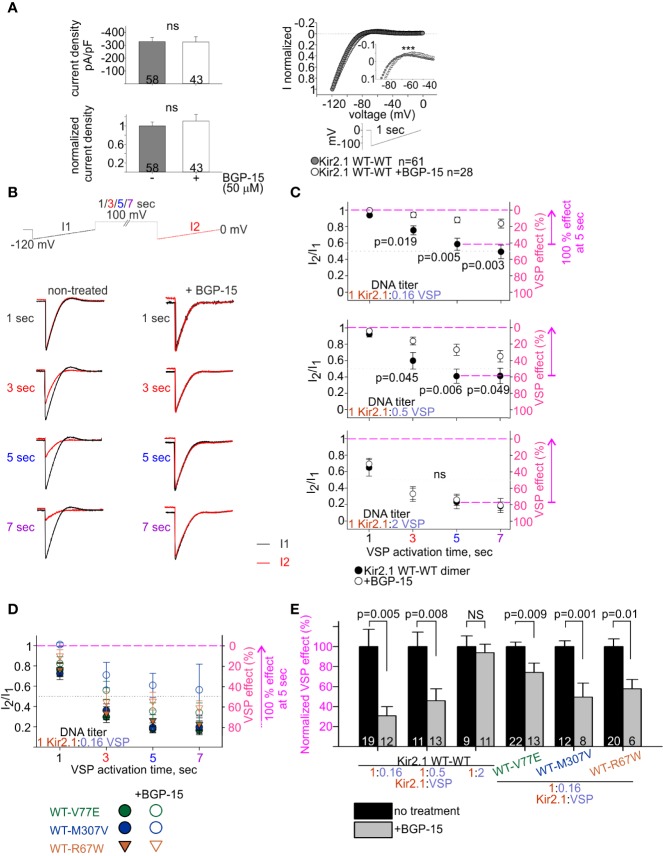
BGP-15 reduces the sensitivity of mutant dimers to PIP_2_ depletion using the calibrated VSP activation protocol. **(A)** Left, averaged current densities at −120 mV from HEK cells expressing Kir2.1 WT dimers with and without BGP-15 treatment. Top, current densities, bottom, current densities in cells that were incubated with BGP-15 and normalized to the mean current density in non-treated cells from the same experiment. Statistical analysis: Mann–Whitney rank-sum test. Right, ramp current waveform normalized to the current at −120 mV, from non-treated cells (same as WT dimer in **Figure 4B**), and BGP-15 treated cells. Statistical analysis: two-way ANOVA with repeated measures, p < 0.001. **(B)** Representative currents elicited in non-treated and 50 µM BGP-15 incubated HEK cells. Example in cells transfected with Kir2.1 WT dimer and VSP in 1:0.5 DNA ratio. **(C)** Average current amplitude ratio and the corresponding VSP effect of WT dimer and VSP expressing cells, following BGP-15 treatment. Top, n = 13, 12, 12, 11 for BGP-15-treated and n = 25, 21, 19, 15 for non-treated cells. N = 3 experiments. Middle, n = 16, 13, 13, 13 for BGP-15-treated and n = 17, 14, 11, 10 for non-treated cells. N = 2 experiments. Bottom, n = 12, 11, 11, 5 for BGP-15-treated and n = 13, 10, 9, 7 for non-treated cells. N = 2 experiments. Statistical analysis: t-test comparing treated with non-treated groups in the same protocol. **(D)** Average current amplitude ratio and the corresponding VSP effect of mutant dimer and VSP expressing cells with and without BGP-15 treatment. Kir2.1:VSP DNA ratio was 1:0.16. VSP effect from non-treated mutant dimers is the same as in **Figure 6B**. For BGP-15 treated groups, WT-V77E n = 18,16,13,7 from N = 3 experiments, WT-M307V n = 5,7,8,3 from N = 1 experiment, WT-R67W n = 7, 8, 6, 6 from N = 2 experiments. **(E)** Normalized VSP effect on Kir2.1 currents following a 5-s depolarization step to 100 mV. The mean VSP effect in non-treated cells (represented by dashed magenta lines in **(C, D)**, of each experiment, is the normalizing value for the VSP effect in panel **(E)**. Statistical analysis: t-test.

We studied the effect of BGP-15 on Kir2.1 current. BGP-15 has been previously shown to have cardio-protective properties and to reduce cardiac arrhythmias, possibly *via* lipid regulation (Gombos et al., 2011; Zhang et al., 2011; Gungor et al., 2014; Sapra et al., 2014). We hypothesized that BGP-15 affects lipid-regulated ion channels’ function, consequently leading to an improvement in cardiac muscle function and reducing cardiac arrhythmia. HEK cells expressing Kir2.1 dimers and VSP were incubated overnight with BGP-15 (50 µM) and currents were recorded on the following day. Kir2.1 current density was not affected by BGP-15 incubation (**Figure 7A**, left). Selectivity and rectification of Kir2.1 current were barely affected by BGP-15 incubation, as shown by the normalized current waveform elicited by a voltage-ramp (**Figure 7A**, right).

We then examined the effect of BGP-15 incubation on the PIP_2_ sensitivity of Kir2.1 WT dimers, using the VSP activation protocol (**Figure 7B**). Within the dynamic range of VSP action, BGP-15 incubation reduced the VSP effect. After 5 s of depolarization to 100 mV, VSP effect in BGP-15 treated cells decreased from 42% ± 7% to 12% ± 3% at 1:0.16 DNA ratio (**Figure 7C**, top) and from 59% ± 8% to 27% ± 7% at 1:0.5 DNA ratio (**Figure 7C**, middle). Basically, when normalized to the VSP effect without BGP-15 from the same experiment, VSP effect in BGP-15 treated cells was reduced to 30% ± 9% and 45% ± 12%, in cells transfected with Kir2.1:VSP DNA ratios of 1:0.16 and 1:0.5, respectively (**Figure 7E**). However, when the VSP proportion was raised to a DNA ratio of 1:2 Kir2.1:VSP, BGP-15 incubation did not reduce the VSP effect. After 5-s depolarization to 100 mV, VSP effect was 77% ± 8% in untreated cells, and 74% ± 7% in BGP-15 treated cells (**Figure 7C**, bottom), and the normalized VSP effect following BGP-15 incubation was 95% ± 6% (**Figure 7E**). To summarize, within a dynamic response range, BGP-15 incubation reduced the VSP effect and stabilized Kir2.1 amplitudes in the calibrated VSP activation protocol.

The VSP activation protocol was then used to test the effect of BGP-15 incubation on the PIP_2_-sensitivity of Kir2.1 mutant dimers. Within the dynamic Kir2.1:VSP DNA ratio of 1:0.16 determined for WT dimers (**Figure 7C**), BGP-15 treatment reduced the VSP effect and increased its variability in the three mutant dimers, compared to the non-treated cells (**Figure 7D**). After 5-s depolarization to 100 mV, the normalized VSP effect following BGP-15 incubation was 74% ± 9%, 49% ± 14%, and 58% ± 9% of the corresponding non treated groups, for the mutant dimers WT-V77E, WT-M307V, and WT-R67W, respectively (**Figure 7E**).

In all, in this set of experiments BGP-15 incubation reduced Kir2.1 current susceptibility to PIP_2_ depletion and stabilized Kir2.1 amplitudes in the calibrated VSP activation protocol, of both Kir2.1 WT and the ATS-associated mutant dimers.

## Discussion

In this study, we report on ATS associated with two mutations in *KCNJ2*, both induce a loss-of-function and exert a dominant-negative effect on the Kir2.1 tetramer. The major outcomes and findings include 1) development of a calibrated functional assay to assess PIP_2_ sensitivity of Kir2.1 current that was tested by VSP activation, and 2) Kir2.1 mutants V77E, M307V, and R67W showed high sensitivity to PIP_2_ depletion, which we interpret as lability in the PIP_2_-Kir2.1 interaction, 3) BGP-15 incubation decreased the sensitivity to PIP_2_ depletion, of both WT and mutant Kir2.1, possibly stabilizing the Kir2.1 open state.

### Phenotypic Variability in ATS

ATS is a rare genetic disease, with a prevalence of 0.08–0.1:100,000 (Horga et al., 2013; Stunnenberg et al., 2018). Cardiac ventricular arrhythmias may include prolonged QT interval, prominent U-wave or ventricular ectopy. Bidirectional VTs were also reported with ATS, although clinical distinction from CPVT is challenging in the absence of other characteristics (Kimura et al., 2012; Chakraborty et al., 2015; Nguyen and Ferns, 2018).

Mutations in Ca^2+^-handling proteins contribute to the etiology of CPVT, while impaired activity of inward-rectifier K^+^-channels is the main cause of ATS (Napolitano et al., 2004; Veerapandiyan et al., 2004). In our report, identification of loss-of-function *KCNJ2* variants established an ATS-1 diagnosis. Although ATS-1 is considered a relatively benign channelopathy, some affected members had malignant ventricular arrhythmias that necessitated an ICD implantation.

The mode of inheritance of the familial M307V mutation was autosomal dominant. Expression was variable and gender-dependent: from six affected family members, three females reported palpitations and presented with incessant bidirectional VT. Two of them had symptomatic arrhythmia events and were implanted with an ICD. In contrast, the three males presented with PVCs, all with normal QT interval, and remained asymptomatic (Nof et al., 2004). The variant M307V was previously reported a *de novo KCNJ2* mutation in a 15-year-old male with ATS manifestations including muscular weakness and dysmorphic features, although his ECG findings were within a normal range (Liu et al., 2015). The V77E mutation occurred *de-novo*, as reported in 30-50% of *KCNJ2* mutations (Tristani-Firouzi et al., 2002; Hasegawa et al., 2015).

### Structural Analysis of the Mutated Kir2.1

**Figure 8:**
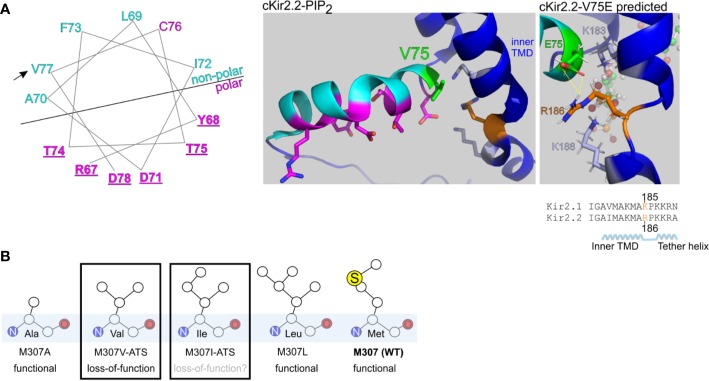
Structural predictions of ATS-associated Kir2.1 variants. **(A)** Left, wheel plot illustration of the residues in Kir2.1 N-terminal slide helix. Polar residues in magenta, non-polar residues in cyan. Mutations in the underlined residues are disease-associated (**Table 1**). Arrow points on the ATS-associated residue in this report. Middle, polar (magenta) and non-polar (cyan) residues in chicken Kir2.2 + PIP_2_ structure (PDB ID:3SPI). V75 in Kir2.2 (green) is the homolog of V77 in Kir2.1. Side chains of K183, K188 (light blue) and R186 (orange) are illustrated. Right, V75E mutation was introduced in Kir2.2 + PIP_2_ structure and underwent a structural refinement. Putative interaction with R186 is shown in yellow. **(B)** Functional properties of Kir2.1 variants in position 307.

The mutation V77E, located in the amphipathic slide helix of Kir2.1 N-terminus, is a substitution of a non-polar, hydrophobic, residue with a negatively charged residue. Most disease-associated variants in Kir2.1 slide helix were detected in polar residues facing the CTD (**Table 1**, **Figure 8A**). Inter and intra-subunit interactions in the interface of CTD and polar residues in the slide helix were suggested to transduce Kir2.1 opening (Decher et al., 2007; Bavro et al., 2012). The position of V77 side chain, in vicinity to the PIP_2_ docking site and particularly the switch to a charged residue, may be implicated in the formation of a new molecular interaction that may have altered the channel interaction network and affected the activation dynamics or Kir2.1-PIP_2_ docking, directly or indirectly. Positively charged residues K182, K185 and K187 on Kir2.1 inner TMD and the tether helix structure (equivalent to K183, R186, and K188 in Kir2.2), are candidates for such interaction. These residues directly interact with 5′ phosphate in the inositol ring of PIP_2_ in a Kir2.2-PIP_2_ structural model (Hansen et al., 2011; Lu et al., 2016).

**Table 1:** Missense variants in Kir2.1 slide helix.

Kir2.1 variant	Diagnosed disease	Reference
R67Q, R67W	ATS, long QT, periodic paralysis	(Eckhardt et al., 2007; Haruna et al., 2007; Andelfinger et al., 2002; Luo et al., 2019)
Y68D	ATS	(Davies et al., 2005)
A70S, A70T	Uncertain significance	ExAc, gnomAD
D71N, D71V, D71Y, D71H, D71E	ATS, long QT, periodic paralysis	(Plaster et al., 2001; Donaldson et al., 2003; Marrus et al., 2011; Luo et al., 2019), LOVD, ClinVar
I72M	Uncertain significance	ExAc, gnomAD
T74A	ATS	(Zhang et al., 2005)
T75M, T75A, T75R	ATS	(Donaldson et al., 2003; Fodstad et al., 2004; Davies et al., 2005)
C76G	Conflicting interpretations of pathogenicity	ClinVar
D78H, D78G, D78Y	ATS, long QT	ClinVar (Davies et al., 2005; Yoon et al., 2006)

ClinVar database obtained the data here from the genetic testing companies Blueprint Genetics, Invitae. and GeneDx.

LOVD, Leiden Open Variation Database, https://www.lovd.nl/; ExAc, The Exome Aggregation Consortium, http://exac.broadinstitute.org/; gnomAD, The Genome Aggregation Database, https://gnomad.broadinstitute.org; ATS, Andersen–Tawil syndrome.

We performed a limited structural analysis of Kir2.1 valine 77 substitution to glutamic acid, based on Kir2.2-PIP_2_ crystal structure (PDB ID: 3SPI) where the corresponding residue is valine 75. The mutated residue E75 formed a putative polar contact with the residue R186 in Kir2.2, which is homologous to K185 in Kir2.1, a residue between the inner TMD and the tether helix (**Figure 8A**, right). Following the energy minimization simulation, we have seen that the R186 side chain is expected to rotate and face the E75 side chain, with a minimal distance of 1.9 Å between the charged residues. This interaction may reduce the flexibility of the tether helix or destabilize the interaction with the agonist PIP_2_, consequently leading to the observed loss-of-function in Kir2.1-V77E.

Methionine 307 is located at the G-loop, a cytoplasmic gating structure in the apex of the CTD (Pegan et al., 2005) (**Figure 1C**). The functional role of the G-loop, its conformation in different gating states and its reciprocated modulation by Kir ligands remain incompletely understood (Pegan et al., 2006; Nishida et al., 2007; Whorton and Mackinnon, 2011; Whorton and Mackinnon, 2013). However, alterations in the pore diameter, shape and biochemical properties conveyed by the G-loop residues’ side chains are expected to lead to functional impairment (Ma et al., 2007). Our functional results show that unlike in the loss-of-function variant Kir2.1-M307V, currents measured in Kir2.1-M307L were indistinguishable from Kir2.1-WT (**Figure 2**), like in the previously reported Kir2.1 variant M307A (Pegan et al., 2005). However, the mutation M307I was found in ATS patients, presumably inducing Kir2.1 loss-of-function (Choi et al., 2007). Remarkably, the size of the hydrophobic side chain did not reflect a structural/functional tolerance (**Figure 8B**), and the potential of methionine to form disulfide bonds seems redundant. Further investigation is needed to shed light on the role of M307 residue, and G-loop in general, in the transition to the open state of Kir2.1.

### Applying the Calibrated VSP Activation Protocol to Study PIP_2_ Regulation of Kir2.1

PIP_2_ is Kir2 primary agonist (Rohacs et al., 2003) and VSP activation reduces Kir2.1 current amplitude by depleting available PIP_2_ molecules at the plasma membrane. We designed a protocol that correlated the changes in Kir2.1 amplitude ratios induced by a calibrated VSP activation, in two heterologous expression systems. We propose that the rate of Kir2.1 current reduction following VSP-induced PIP_2_ depletion reflects Kir2.1 sensitivity to PIP_2_ interaction so that an accelerated response rate is the consequence of a lower gating efficiency due to low PIP_2_ affinity to Kir2.1.

In the calibrated VSP activation protocol we compared functional properties of dimer-composed Kir2.1 channels: the homomeric WT channel and the three heteromeric Kir2.1 mutants. We assumed that two dimers assembled into a tetrameric protein, but in a less-likely scenario four WT subunits originated from four mutated dimers might have assembled into one functional homomeric WT channel. This scenario cannot explain the increase in the VSP effect that occurred in mutant dimers but not in WT dimers (**Figure 6**).

The three Kir2.1 mutants putatively represent three mechanisms of Kir2.1 loss-of-function: V77E that destabilizes the tether helix, M307V that affects G-loop structure and R67W that is involved in inter-subunit interaction with the tether helix and the CTD. Yet, the three Kir2.1 mutant dimers showed a similar reduction in the amplitude compared to the WT dimer (**Figure 4**), and the calibrated VSP activation protocol did not reveal a difference in the VSP effect of the three mutant dimers (**Figure 6**), in two expression systems. We propose that the mechanisms of the pathogenic variants V77E, M307V, and R67W converge onto a similar impairment in the gating mechanism. One potential mechanism is a reduced affinity to the agonist PIP_2_ in mutated Kir2.1 subunits and a total reduction in PIP_2_ interaction in the heteromeric channel that destabilizes an open state. Based on available crystal structures, we propose a direct impairment of PIP_2_ interaction in the V77E variant (**Figure 8**) and an allosteric effect in R67W and M307V variants that may impair an open state conformational transduction.

Finally, we tested the effect of BGP-15 on Kir2.1 currents and its therapeutic potential in ATS-associated Kir2.1 mutants. In a previous report, the oral administration of BGP-15 reduced AF episodes in genetically-engineered mice (Sapra et al., 2014). The AF was induced by age-dependent electrical remodeling that accompanied atrial enlargement in two transgenic mouse models with a dilated and failing heart. The AF mouse model had reduced ion channel expression, including *KCNJ3* (Kir3.1) and *KCNA5* (Kv1.5) genes (Sapra et al., 2014). Normalization of the fibrillation episodes by BGP-15 may have been related to the stabilization of the resting potential by an increased activity of potassium channels. In this study, following BGP-15 incubation, Kir2.1 currents were more resistant to PIP_2_ depletion by VSP activation. We propose that BGP-15 is associated with the regulation of PIP_2_ level, availability or localization, resulting in stabilization of Kir2.1 open-state. An indirect effect on other lipid regulators of Kir2.1 is plausible, e.g. *via* molulation of the interaction with Kir2.1 suppresor cholesterol, as BGP-15 was shown to remodel cholesterol-enriched lipid platforms (Romanenko et al., 2004; Gombos et al., 2011).

To note, Kir2.1 is predominantly expressed in the cardiac ventricles and ATS is an electrophysiologic disorder of the ventricle (Melnyk et al., 2002). However, other PIP_2_-dependent inward rectifier potassium channels are expressed in the atria, mainly Kir2.2, Kir2.3, and acetylcholine-activated Kir3.1 and Kir3.4, in addition to other potassium channels such as Kv11.1 (hERG), Kv7.1 (KCNQ1), and Kir6.2 (Schram et al., 2002). Further investigation will shed light on whether the BGP-15 effect is shared by other PIP_2_-dependent ion channels or is unique to Kir2.1.

## Conclusion

In this study, two cases of ATS with cardiac arrhythmias were described, highlighting ATS phenotypic variability and resemblance with CPVT. The ATS-associated mutations, V77E and M307V, expand the spectrum of known *KCNJ2* mutations. Mutations were functionally and biochemically studied and showed loss-of-function with a dominant-negative effect. A calibrated protocol quantitated Kir2.1 current amplitude sensitivity to PIP_2_ depletion and was tested on ATS-associated variants. The Kir2.1 variants showed increased PIP_2_ sensitivity, which was partially restored by BGP-15.

## Data Availability Statement

The datasets generated for this study can be found in the LOVD [https://databases.lovd.nl/shared/variants/0000630833].

## Author Contributions 

RH-J performed TEVC experiments. EM designed the study and prepared DNA constructs. DY performed simulations. LV performed biochemical studies. RB, MG, and EN evaluated the patients. ND, MG, JM, and SO acquired financial support for the project. ND, JM, and SO conceived and planned the experiments. SO designed the study, performed patch-clamp experiments, and wrote the manuscript with input from all authors.

## Funding 

This work was supported by Dizengoff Trading Company (1952) Ltd. (MG), Israel Science Foundation (1282/18; ND), and Melbourne Biomedical Precinct Grant, Victorian Government’s Trade and Investment Office for Israel Funding Program (JM and SO).

## Conflict of Interest

The authors declare that the research was conducted in the absence of any commercial or financial relationships that could be construed as a potential conflict of interest.

The authors declare that this study received funding from Dizengoff Trading Company (1952) Ltd. The funder was not involved in the study design, collection, analysis, interpretation of data, the writing of this article, or the decision to submit it for publication.
