# Supplementary figures and images for: Andersen–Tawil Syndrome Is Associated With Impaired PIP2 Regulation of the Potassium Channel Kir2.1
Source: Front Pharmacol. 2020 May 15;11:672. doi: 10.3389/fphar.2020.00672 (PMC7243181; doi:10.3389/fphar.2020.00672)

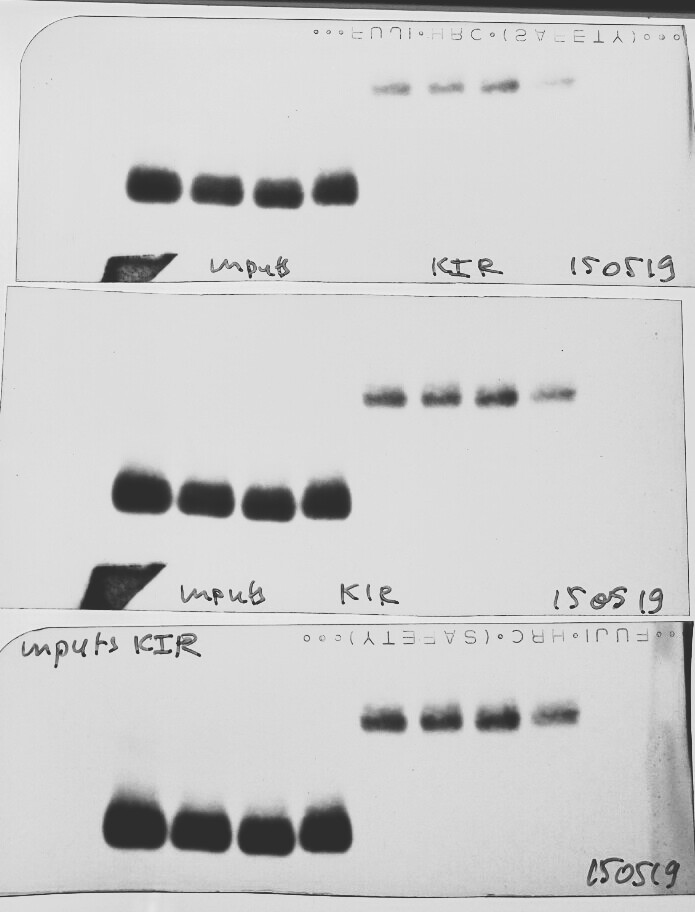

Supplement: Supplementary file 1 [file Image_1.jpeg]

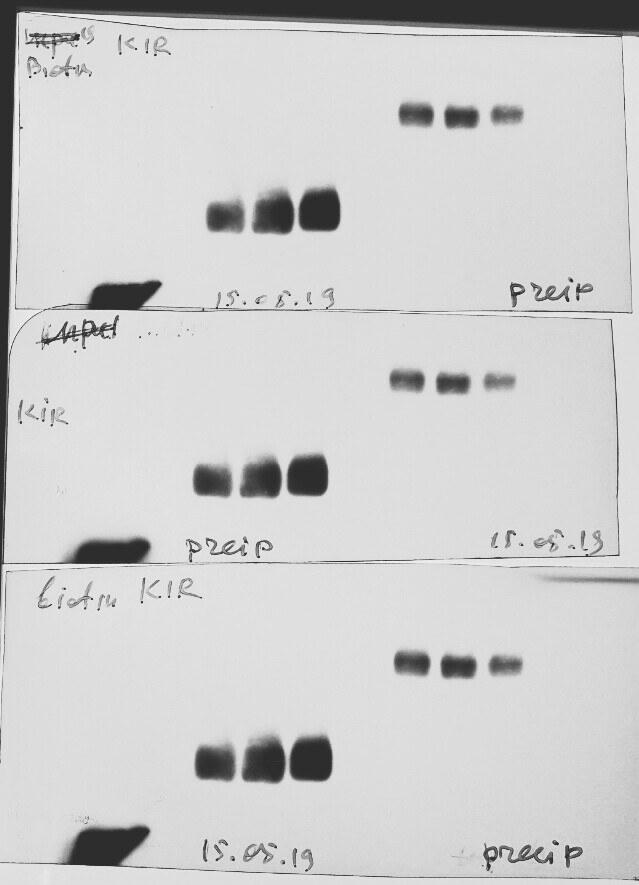

Supplement: Supplementary file 2 [file Image_2.jpeg]
